# GFI1^36N^ as a therapeutic and prognostic marker for myelodysplastic syndrome

**DOI:** 10.1016/j.exphem.2016.04.001

**Published:** 2016-07

**Authors:** Lacramioara Botezatu, Lars C. Michel, Hideki Makishima, Thomas Schroeder, Ulrich Germing, Rainer Haas, Bert van der Reijden, Anne E. Marneth, Saskia M. Bergevoet, Joop H. Jansen, Bartlomiej Przychodzen, Marcin Wlodarski, Charlotte Niemeyer, Uwe Platzbecker, Gerhard Ehninger, Ashwin Unnikrishnan, Dominik Beck, John Pimanda, Eva Hellström-Lindberg, Luca Malcovati, Jacqueline Boultwood, Andrea Pellagatti, Elli Papaemmanuil, Philipp Le Coutre, Jaspal Kaeda, Bertram Opalka, Tarik Möröy, Ulrich Dührsen, Jaroslaw Maciejewski, Cyrus Khandanpour

**Affiliations:** a Department of Hematology, West German Cancer Center, University Hospital Essen, University Duisburg-Essen, Essen, Germany; b Department of Translational Hematology and Oncology Research, Taussig Cancer Institute, Cleveland, OH, USA; c Department of Hematology and Oncology, University Hospital Düsseldorf, Düsseldorf, Germany; d Department of Laboratory Medicine, Radboud University Nijmegen Medical Centre, Nijmegen, The Netherlands; e Division of Pediatric Hematology and Oncology, Department of Pediatrics and Adolescent Medicine, University of Freiburg, Freiburg, Germany; f Department of Internal Medicine I, University Hospital TU Dresden, Dresden, Germany; g Lowy Cancer Research Centre and Prince of Wales Clinical School, University of New South Wales, Sydney, Australia; h Center for Hematology and Regenerative Medicine, Karolinska University Hospital Huddinge, Stockholm, Sweden; i Department of Molecular Medicine, University of Pavia, and Department of Hematology Oncology, Fondazione IRCCS Policlinico San Matteo, Pavia, Italy; j Nuffield Division of Clinical Laboratory Sciences, Radcliffe Department of Medicine, University of Oxford, Oxford, United Kingdom; k Cancer Genome Project, Wellcome Trust Sanger Institute, Hinxton, Cambridge, United Kingdom; l Medical Department with Focus on Hematology/Oncology Charite Berlin, Campus Virchow-Klinikum, Berlin, Germany; m Institut de Recherches Cliniques de Montréal (IRCM), Hematopoiesis and Cancer Research Unit, and Département de Microbiologie, Infectiologie et Immunologie, Université de Montréal, Montréal, Canada

## Abstract

Inherited gene variants play an important role in malignant diseases. The transcriptional repressor growth factor independence 1 (GFI1) regulates hematopoietic stem cell (HSC) self-renewal and differentiation. A single-nucleotide polymorphism of GFI1 (rs34631763) generates a protein with an asparagine (N) instead of a serine (S) at position 36 (GFI1^36N^) and has a prevalence of 3%–5% among Caucasians. Because GFI1 regulates myeloid development, we examined the role of GFI1^36N^ on the course of MDS disease. To this end, we determined allele frequencies of GFI1^36N^ in four independent MDS cohorts from the Netherlands and Belgium, Germany, the ICGC consortium, and the United States. The GFI1^36N^ allele frequency in the 723 MDS patients genotyped ranged between 9% and 12%. GFI1^36N^ was an independent adverse prognostic factor for overall survival, acute myeloid leukemia-free survival, and event-free survival in a univariate analysis. After adjustment for age, bone marrow blast percentage, IPSS score, mutational status, and cytogenetic findings, GFI1^36N^ remained an independent adverse prognostic marker. GFI1^36S^ homozygous patients exhibited a sustained response to treatment with hypomethylating agents, whereas GFI1^36N^ patients had a poor sustained response to this therapy. Because allele status of GFI1^36N^ is readily determined using basic molecular techniques, we propose inclusion of GFI1^36N^ status in future prospective studies for MDS patients to better predict prognosis and guide therapeutic decisions.

## Highlights

• GFI1^36N^ is present in about 9%–12% of all Caucasian patients with myelodysplastic syndrome.
• GFI1^36N^ is an independent, adverse prognostic factor for survival.
• GFI1^36N^ patients with myelodysplastic syndrome respond poorly to hypomethylating agents.

## Graphical abstract

**Figure:**
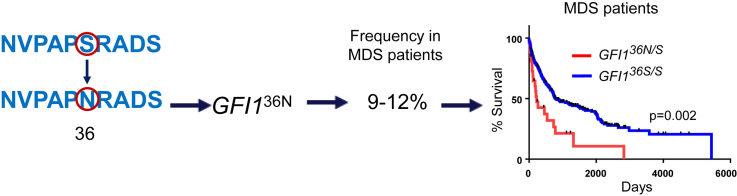

GFI1 is a zinc finger transcriptional repressor that recruits histone-modifying enzymes, such as histone deacetylases, to the loci of its target genes 1, 2. GFI1 regulates the functions of hematopoietic stem cells (HSCs) 1, 3 as well as myeloid–lymphoid lineage decisions 4, 5. A variant form of the GFI1 gene (denominated GFI1^36N^) is associated with a predisposition to develop de novo acute myeloid leukemia (AML) [6], but it has also been reported to be involved in a case of neutropenia [7]. Taking into consideration the predisposing role of GFI1^36N^ to de novo AML and its role in myeloid development, we investigated the role of GFI1^36N^ in myelodysplastic syndrome (MDS).

## Methods

### Patient cohort

All patient samples (peripheral blood [PB] or bone marrow [BM] aspirates) were obtained with informed consent according to the Declaration of Helsinki. The respective local ethics committees approved the use of all patient samples.

The clinical characteristics of patients with a confirmed diagnosis of MDS used in this study have been previously described 8, 9, 10, 11, 12, 13. Events in “event-free survival” were defined as death from any cause or progression of MDS to AML with blast counts higher than 20%. Overall survival events are defined as death from any cause.

### Bone marrow morphology and cytopenia classification

Bone marrow morphology studies were performed at individual centers. MDS was classified based on the World Health Organization (WHO) definition [14].

### Genotyping

Genotyping was performed according to published procedures [6].

### Statistical methods

Significance of differences in percentages was determined using the two-sided, two-sample *t* test. Survival of the different human cohorts is based on the presence of GFI1^36N^ univariate analysis using Kaplan–Meier survival methods. Differences were assessed using the log-rank (Mantel–Cox) test. We used Cox proportional-hazards regression modeling to determine the influence of different factors with respect to survival. Factors taken into account were International Prognostic Scoring System (IPSS) risk group, BM blast count, age, sex, cytogenetic findings (based on IPSS classification), and in a last step, presence of GFI1^36N^. Analyses were performed either separately (with each factor analyzed independently) or with the presence or absence of GFI1^36N^. All *p* values reported are two-sided. Because of the exploratory nature of this study, no adjustment for multiple testing was done. All analyses presented were performed using GraphPad Prism 6 software (GraphPad Software, La Jolla, CA) or SPSS Version 19 (IBM, Armonk, NY).

## Results

Between 9% and 12% of all adult MDS patients in four different Caucasian cohorts from Europe and the United States 8, 9, 10, 11, 12 were heterozygous for GFI1^36N^, and only two patients from the European cohorts (and none of the U.S. cohort) were homozygous for GFI1^36N^ (Table 1). GFI1^36N^ allele frequency was higher among MDS patients than among the control cohort (3%–5%) as reported in our previous study regarding the role of GFI1^36N^ in de novo AML [15]. Although we did not determine the frequency of GFI1^36N^ among sex- and age-matched control subjects from every region, it is possible that GFI1^36N^ predisposes to MDS, similar to its predisposing role in de novo AML [15].

We analyzed the effect of GFI1^36N^ on MDS disease course in two independent cohorts. Patients were recruited and treated either in the United States or in Europe. The European cohorts consisted of patients recruited and treated at different centers in Germany, Belgium, and The Netherlands 8, 9, 10, 11, 12. The U.S. cohorts were referred to the Cleveland Clinic.

**Figure 1:**
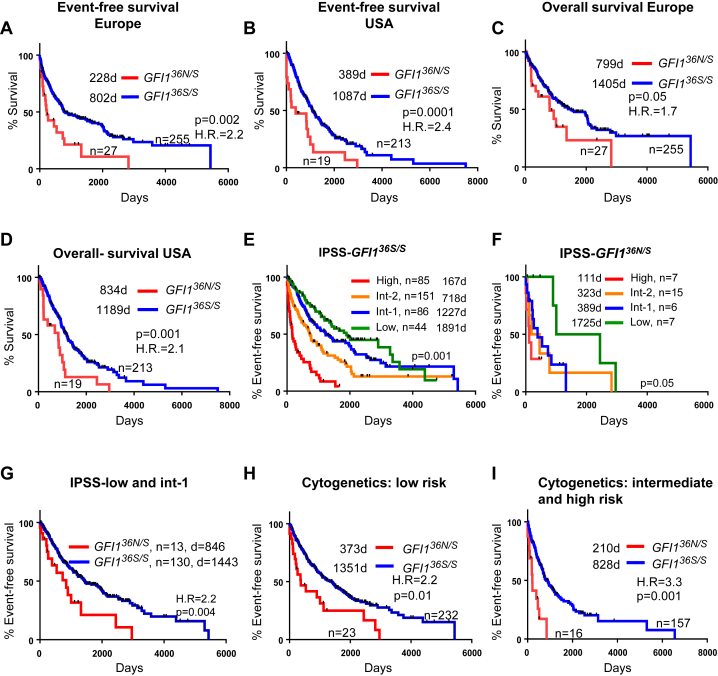
Correlation between GFI1^36N^ and disease course of patients with MDS. (**A**) Patients from different European cohorts diagnosed with MDS on the basis of WHO criteria were genotyped with respect to the presence of GFI1^36N^ and examined with respect to median event-free survival (see also Methods); 95% confidence interval (CI) = 1.6–5.6. Median survival is indicated. (**B**) MDS Patients from a U.S. cohort diagnosed with MDS on the basis of WHO criteria were genotyped with respect to the presence of GFI1^36N^ and examined with respect to median event-free survival; 95% CI = 1.7–7.2. Median survival is indicated. (**C**) The same cohorts as in (**A**) were examined with respect to overall survival (death of any cause); 95% CI = 1.0–3.9. Median survival is indicated. (**D**) The same cohort as in (**B**) was examined with respect to overall survival (death from any cause) 95% CI = 1.5–5.8. Median survival is indicated. (**E**) Event-free survival of GFI1^36S^ homozygous patients was stratified based on IPSS classification. No sufficient follow-up was available for the International Cancer Genome Consortium (ICGC) patients. Follow-up is based on the patient cohorts from the United States, the Netherlands, Belgium, and Germany. Median survival is indicated. (**F**) Event-free survival of GFI1^36N^ homozygous or heterozygous patients was stratified based on IPSS classification. No sufficient follow up was available for the ICGC patients. Follow-up is based on the patient cohorts from the United States, the Netherlands, Belgium, and Germany. Median survival is indicated. (**G**) Event-free survival of patients (shown in A) classified as either IPSS subtype low or intermediate 1 (int-1) was stratified with respect to the presence of GFI1^36N^; 95% CI = 1.4–6.2. (**H**) Patients from the U.S. and European cohorts with cytogenetic risk characteristics belonging to subtype “low” were stratified by the presence of GFI1^36N^ with respect to event-free survival; 95% CI = 1.5–5.7. Median survival is indicated. (**I**) Patients from the U.S. and European cohorts with cytogenetic risk characteristics belonging to subtype “intermediate” or “high” were stratified by presence of GFI1^36N^ with respect to event-free survival; 95% CI = 3.3–23.3. Median survival is indicated.

In the two MDS cohorts from Europe and the United States, presence of GFI1^36N^ was associated with an inferior event-free survival rate (Fig. 1A, B). GFI1^36N^ also had a negative impact on overall survival (Fig. 1C, D) and on AML-free survival (data not shown).

Next, we examined the association between GFI1^36N^ and established prognostic factors. GFI1^36N^ carriers were older, exhibited a tendency toward higher BM blast counts at diagnosis, were diagnosed with a more advanced stage of the disease according to histologic parameters, and had more adverse cytogenetic findings (Table 1). With respect to key blood parameters, no other differences between GFI1^36N^ and GFI1^36S^ carriers were observed (Table 1).

American and European GFI1^36S^ homozygous patients had median follow-ups of 1,100 and 975 days, respectively. American and European GFI1^36N^ carriers had median follow-ups of 540 and 350 days, respectively. To gain more statistical power and to perform more specific analysis, we combined the U.S. and European cohorts. One approach used to predict outcome of MDS patients is based on IPSS 16, 17. A recently introduced, revised version of IPSS (denominated IPSS-R) distinguishes between more subclasses based on cytogenetic findings and cytopenic lineages [16]. However, not all of the specific data for determining IPSS-R status were present in our databases. Therefore, we focused our examination on the nonrevised version of IPSS.

As reported previously [17], IPSS scoring predicted event-free outcome of GFI1^36S^ homozygous patients (Fig. 1E). On the basis of the same scoring system, GFI1^36N^ carriers had a significantly shorter event-free survival (Fig. 1F). Especially among MDS patients in the low-risk groups (groups low and intermediate 1 based on IPSS), GFI1^36N^ carriers had a much shorter event-free (Fig. 1G) and AML-free survival (data not shown) than GFI1^36S^ homozygous patients. We also examined the association between allele status, cytogenetic findings and event-free survival. Similarly, presence of GFI1^36N^ was associated with an inferior outcome, independent of the cytogenetic finding (Table 1). After stratification for cytogenetic risk groups (“low” as one group and “intermediate and high” as a second group), the presence of a GFI1^36N^ allele was again linked to inferior event-free survival (Fig. 1H, I) in both comparisons.

**Figure 2:**
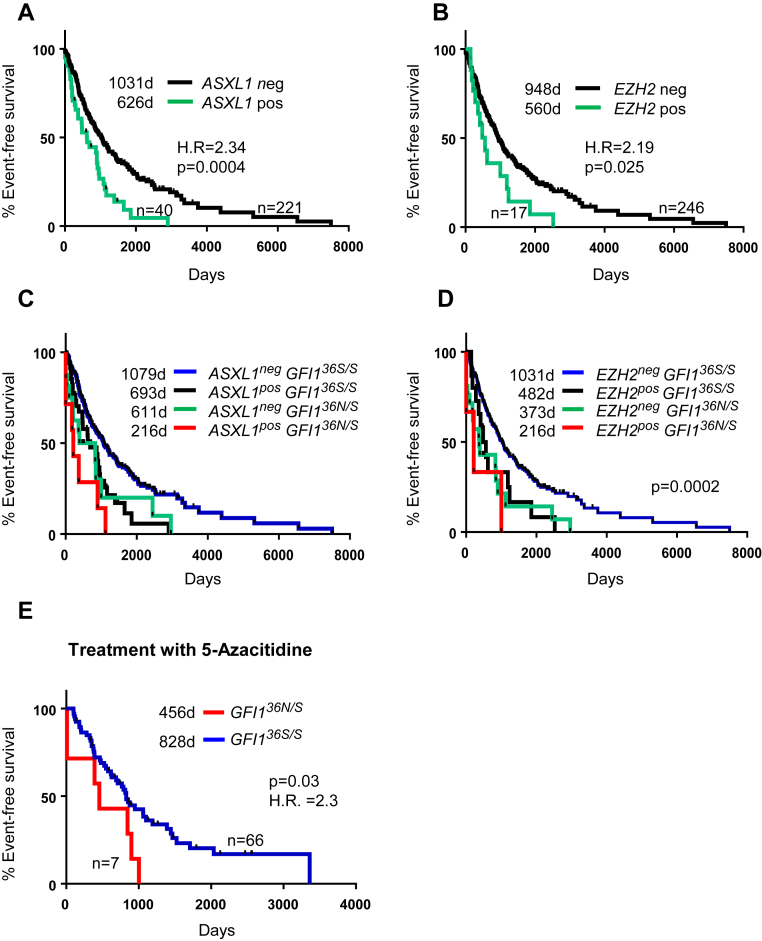
Association between GFI1^36N^, mutational status, prognosis, and therapeutic response. (**A**) Median event-free survival of patients based on mutational status of ASXL1; 95% confidence interval (CI) = 1.5–5.7. (**B**) Median event-free survival of patients based on mutational status of EZH2; 95% CI = 3.4–23.3. (**C**) Median event-free survival of patients based on presence of GFI1^36N^ and mutational status of ASXL1. (**D**) Median event-free survival of patients based on presence of GFI1^36N^ and mutational status of EZH2. (**E**) From the cohorts of patients treated in Europe and the United States, patients treated with 5-azacitidine were stratified with respect to GFI1 status. Presence of GFI1^36N^ was associated with a shorter response. Median survival is indicated; 95% CI = 1.1–10.5.

MDS patients with somatic mutations within EZH2 or ASXL1 have an inferior prognosis 18, 19 (Fig. 2A, B). Presence of GFI1^36N^ in the absence of ASXL1 or EZH2 mutations had a similar effect on event-free survival as the presence of ASXL1 or EZH2 mutations (Fig. 2C, D). Furthermore, the combined presence of a mutated form of either ASXL1 or EZH2 and a GFI1^36N^ allele had an additional adverse effect on event-free survival (Fig. 2C, D). Similar analyses were not possible for mutations of P53 or RUNX1 18, 19, because of the small number of cases with P53 or RUNX1 mutation and GFI1^36N^.

Because MDS patients who are hetero- or homozygous for GFI1^36N^ tend to be older at diagnosis, have more frequent adverse cytogenetic findings, and have a higher blast cell count at diagnosis, we examined whether the presence of GFI1^36N^ represents an adverse prognostic factor after adjusting for these findings. We found that GFI1^36N^ was an independent marker after adjusting for the variables IPSS score, cytogenetic findings, and age, either alone or in combination (Supplementary Table E1, Supplementary Table E2, online only, available at www.exphem.org).

## Discussion

One possible explanation why GFI1^36N^ accelerates AML development in MDS patients could be based on our findings using murine models. We previously generated mice expressing GFI1^36N^ or GFI1^36S^ instead of murine Gfi1 [15]. We reported that GFI1^36N^ is not able to bind to its target genes to the same extent as the more common “wild-type” GFI1^36S^ variant. Hence, presence of one allele of GFI1^36N^ led to higher genomewide levels of acetylated histone 3 at lysine 9 (H3K9) at Gfi1 target genes. This led to active expression of genes favoring development of myeloid malignancies [15], which could explain how GFI1^36N^ accelerates both AML development and MDS–AML progression. We recently reported that a low level of GFI1 expression, which might mimic the presence of GFI1^36N^ on a functional level, accelerates AML progression in different murine models of AML, including one model of MDS [20]. It is not yet clear why altering one amino acid changes the function of GFI1, and initial experiments regarding expression level, stability, ability to induce apoptosis, or interaction with histone deacetylases (HDACs) or lysine-specific demethylase 1 (LSD1) did not reveal any significant differences between GFI1^36N^ and GFI1^36S^ [15] (unpublished data).

We also examined whether GFI1^36N^ could predict response to therapy. To explore this in more detail, we focused on patients that were treated with 5-azacitidine. This treatment is used for patients who are otherwise not fit for allogeneic bone marrow transplantation or as a bridging to a definitive therapy 21, 22, 23. There was no difference between GFI1^36N^ heterozygous carriers and GFI1^36S^ patients achieving response to treatment with 5-azacytidine (51% of GFI1^36S^ homozygous carriers compared with 52% of GFI1^36N^ carriers). However, the response to treatment was much shorter in GFI1^36N^ carriers than in GFI1^36S^ homozygous carriers (Fig. 2E). This observation is not surprising because treatment with hypomethylating agents, such as 5-azacitidine, would not revert the increased levels of H3K9 acetylation and H3K4 methylation seen in cells with a GFI1^36N^ allele [15].

The suitability of GFI1^36N^ as a prognostic marker should be verified in prospective studies and, if the findings can be confirmed, the status of GFI1^36N^ should be determined routinely in MDS patients. Considering the role of GFI1 in myeloid development, GFI1^36N^ status could also be of prognostic value for patients with myeloproliferative diseases and chronic myeloid leukemia. Indeed, the frequency of GFI1^36N^ is also elevated among patients with chronic myeloid leukemia. In summary, GFI1^36N^ could be a useful therapeutic and prognostic marker for myeloid malignancies.

## Figures and Tables

**Supplementary Table E1:** Cox survival regression adjusted for IPSS^a^

Variable	Wald χ^2^	*p* value	Hazard ratio	95% CI for hazard ratio	
Lower	Upper
IPSS	77.381	0.000			
IPSS low	13.699	0.000	0.504	0.350	0.724
IPSS intermediate 1	4.607	0.032	0.725	0.540	0.972
IPSS intermediate 2	1.586	0.208	1.229	0.892	1.694
IPSS high	32.297	0.000	3.158	2.124	4.696
GFI1^36N^	20.125	0.000	2.212	1.564	3.130

CI = confidence interval.

a IPSS was defined in four different entities as published (for details, see main text).

**Supplementary Table E2:** Cox survival regression adjusted for key prognostic factors^a^

Variable	Wald χ^2^	*P* value	Hazard ratio	95.0% CI for hazard ratio	
Lower	Upper
Age	4.761	0.029	1.010	1.001	1.019
Blast (% BM)	7.141	0.008	1.045	1.012	1.078
Male	0.518	0.472	1.111	0.834	1.479
IPSS	5.682	0.224			
IPSS low	5.081	0.024	0.571	0.351	0.930
IPSS intermediate 1	2.561	0.110	0 .728	0.494	1.074
IPSS intermediate 2	0.205	0.651	0.909	0.603	1.371
IPSS high	0.088	0.767	1.105	0.570	2.144
Cytogenetic	12.264	0.007			
Cytogenetic good	0.115	0.735	0.894	0.466	1.713
Cytogenetic intermediate	1.089	0.297	0.686	0.339	1.392
Cytogenetic poor	1.278	0 .258	1.469	0.754	2.862
GFI1^36N^	10.720	0.001	2.154	1.361	3.409

CI = confidence interval.

a Cox survival regression adjusted for age, blast count, sex, IPSS, cytogenetic finding, and as a last step, presence of GFI136N (see main text for more information).

**Table 1:** Features of GFI1^36N^- and GFI1^36S^-carrying adult MDS patients^a^

	GFI1^36N^ (homo [2 patients]- or heterozygous)	GFI1^36S^ (homozygozus)	*P* value
% All countries	10 (*n* = 75)	90 (*n* = 648)	
% United States	11 (*n* = 30)	89 (*n* = 254)	
% Germany	9 (*n* = 20)	91 (*n* = 193)	
% Netherlands and Belgium	12 (*n* = 11)	88 (*n* = 84)	
% ICGC	11 (*n* = 14)	89 (*n* = 117)	
Mean age	66 ± 1.5 (*n* = 73)	62 ± 0.6 (*n* = 638)	0.01
Gender (% male)	64 (*n* = 47)	66 (*n* = 426)	0.7
Blast percentage (BM) WHO	9.4 ± 0.3 (*n* = 48)	6.7 ± 1.1 (*n* = 464)	0.01
Hemoglobin (mg/dL)	8.4 ± 0.5 (*n* = 24)	8.1 ± 0.1 (*n* = 191)	0.57
Platelet count (1/nL)	186 ± 37 (*n* = 24)	177 ± 11 (*n* = 195)	0.8
Neutrophil count (1/nL)	2.8 ± 0.8 (*n* = 24)	2.7 ± 0.2 (n = 196)	0.9
Cytogenetic low risk (%)	46 (*n* = 27)	62 (*n* = 246)	0.03
Cytogenetic intermediate risk (%)	14 (*n* = 8)	16 (*n* = 64)	0.5
Cytogenetic high risk (%)	41 (*n* = 24)	24 (*n* = 96)	0.006
IPSS low (%)	25 (*n* = 13)	25 (*n* = 127)	0.7
IPSS intermediate 1 (%)	40 (*n* = 38)	42 (*n* = 323)	0.7
IPSS intermediate 2 (%)	21 (*n* = 11)	21 (*n* = 109)	0.7
IPSS high (%)	15 (*n* = 8)	10 (*n* = 52)	0.25
5q– (%)	5 (*n* = 2)	4 (*n* = 13)	0.8
RA (%)	0 (*n* = 0)	9 (*n* = 30)	0.054
RARS+ RARST (%)	11 (*n* = 4)	9 (*n* = 28)	0.7
RAEB-1 (%)	22 (*n* = 8)	18 (*n* = 59)	0.65
RAEB-2 (%)	39 (*n* = 15)	22 (*n* = 69)	0.02
RAEB-1 + RAEB-2 (%)	61 (23)	40 (128)	0.01
RCMD (%)	24 (*n* = 9)	38 (*n* = 123)	0.09
MDS-u (%)	0	1 (*n* = 4)	0.5

BM = bone marrow; ICGC = International Cancer Genome Consortium; WHO = World Health Organization.

a GFI1^36N^ includes patients who are either homozygous or heterozygous for GFI1^36N^ and, thus, carrying one GFI1^36S^ allele. GFI1^36S^ refers to patients homozygous for GFI1^36S^. Cytogenetic low risk: normal karyotype, 5q–, 20q–, –Y; poor risk: complex aberrations (≥3 anomalies), chromosome 7 anomalies; intermediate risk: all other aberrations. IPSS was based on Greenberg et al. [17]. Refractory anemia (RA), refractory anemia with ring sideroblasts (RARS), refractory anemia with excess blasts, type 1 (RAEB-1), refractory anemia with excess blasts, type 2 (RAEB-2), refractory cytopenia with multilineage dysplasia (RCMD), and MDS-unclassified (MDS-u) are based on the WHO definition for MDS. Standard errors of mean are given. Data for IPPS and cytogenetic classification are missing due to the lack of cytogenetic information on patients at the time of diagnosis. Data for histologic classification are missing because of the missing specification of MDS according to WHO criteria. The missing patients were diagnosed as having MDS according to the WHO definition. Student's *t* test was used to determine the significance of values, and two-sample *t* tests were used to determine the significance of differences between percentages. The different cohorts were independent of each other. No overlap exists with respect to samples. The numbers in parentheses correspond to the absolute numbers related to the indicated percentages.
